# Modulation of *MDR1* and *MRP3* Gene Expression in Lung Cancer Cells after Paclitaxel and Carboplatin Exposure

**DOI:** 10.3390/ijms131216624

**Published:** 2012-12-05

**Authors:** Consolación Melguizo, Jose Prados, Raquel Luque, Raúl Ortiz, Octavio Caba, Pablo J. Álvarez, Beatriz Gonzalez, Antonia Aranega

**Affiliations:** 1 Institute of Biopathology and Regenerative Medicine (IBIMER), Department of Anatomy and Human Embryology, School of Medicine, University of Granada, Granada E-18071, Spain; E-Mails: melguizo@ugr.es (C.M.); pabrolancia@hotmail.com (P.J.Á.); aranega@ugr.es (A.A.); 2 Service of Medical Oncology, Virgen de las Nieves Hospital, Granada E-18012, Spain; E-Mails: rluquecaro@gmail.com (R.L.); bea_astorga@hotmail.com (B.G.); 3 Department of Health Science, University of Jaén, Jaén E-23071, Spain; E-Mails: roquesa@ugr.es (R.O.); ocaba@ugr.es (O.C.)

**Keywords:** lung cancer, resistance, MDR, MRP, paclitaxel, carboplatin

## Abstract

Carboplatin-paclitaxel is a reference regimen in the treatment of locally advanced or disseminated non-small cell lung cancer (NSCLC). This paper discusses the multidrug resistance developed with this drug combination, which is one of the major obstacles to successful treatment. In order to understand and overcome the drug resistance pattern of NSCLC after carboplatin plus paclitaxel exposure, levels of mRNA expression of multidrug resistance 1 (MDR1) and multidrug resistance-associated protein 3 (MRP3) were investigated in primary NSCLC cell lines (A-549 and A-427) and a metastasis-derived NSCLC cell line (NODO). Our results showed that exposure of the three NSCLC lines to plasma concentrations of paclitaxel (5 μM) produced an increase in MDR1 expression, while MRP3 showed no alteration in expression. By contrast, the same cells exposed to carboplatin plasma concentrations (30 μM) showed overexpression of MRP3. In these cells, MDR1 showed no expression changes. Interestingly, the combination of both paclitaxel and carboplatin caused increased expression of the MDR1 drug resistance gene rather than the individual treatments. These results suggest that carboplatin and paclitaxel may induce drug resistance mediated by MDR1 and MRP3, which may be enhanced by the simultaneous use of both drugs.

## 1. Introduction

Non-small cell lung cancer (NSCLC) is currently the leading cause of cancer deaths worldwide. Unfortunately, once this tumor is diagnosed, most patients have locally advanced or disseminated cancers. Comparison of third-generation chemotherapy regimens, which included Carboplatin (Car)-Paclitaxel (Pac), Cisplatin-Pac, Cisplatin-Gemcitabine and Cisplatin-Docetaxel, showed no differences in survival, but Car-Pac showed the lowest degree of toxicity and is therefore the reference regimen in NSCLC patients [1]. However, development of multidrug resistance (MDR) is the main cause of the failure of this chemotherapeutic treatment.

Despite MDR being the primary cause of NSCLC recurrence and metastasis [2], its molecular mechanisms remain poorly understood. P-glycoprotein (P-gp), multidrug resistance protein (MRP) and lung cancer-related protein (LRP) have been implicated in NSCLC drug resistance [3–5]. P-glycoprotein is an ATP-dependent drug efflux protein codified by the gene *MDR1* that transports various structurally unrelated compounds to maintain their intracellular concentrations below cytotoxic levels [4]. Cytotoxic drugs used classically in the treatment of lung cancer, such as platinum-based drugs (cisplatin and Ca), etoposide, doxorubicin and Pac, have been linked to MDR1 expression [6,7]. Multidrug resistance protein (MRP) is a member of the ABC transport protein superfamily. Overexpression of MRP has been detected in a variety of tumor types and is associated with drug resistance with a mechanism similar to P-gp [8]. MRP was previously correlated with human lung cancer [9] and has been involved in drug resistance in NSCLC cell lines [10,11]. Studies of tumor xenografts showed that overexpression of Bcl-2 and MRP3 was one of the major mechanisms of the resistance [12]. Recently, Chen *et al.* showed that LRP plays a role in multidrug resistance in NSCLC [13], although in resistant cell lines, it has been linked to the use of doxorubicin [14].

To date, modulation of resistance-gene expression by chemotherapy regimens in NSCLC patients and their use as prognostic markers is unknown and needs further investigation. Our study examined the modulation of MDR1 and MRP3 gene expression in NSCLC cancer cell lines treated with plasma concentrations of Pac and Car in order to understand the resistance mechanism against these drugs.

## 2. Results

### 2.1. Drug Cytotoxicity in NSCLC Cells

**Figure 1:**
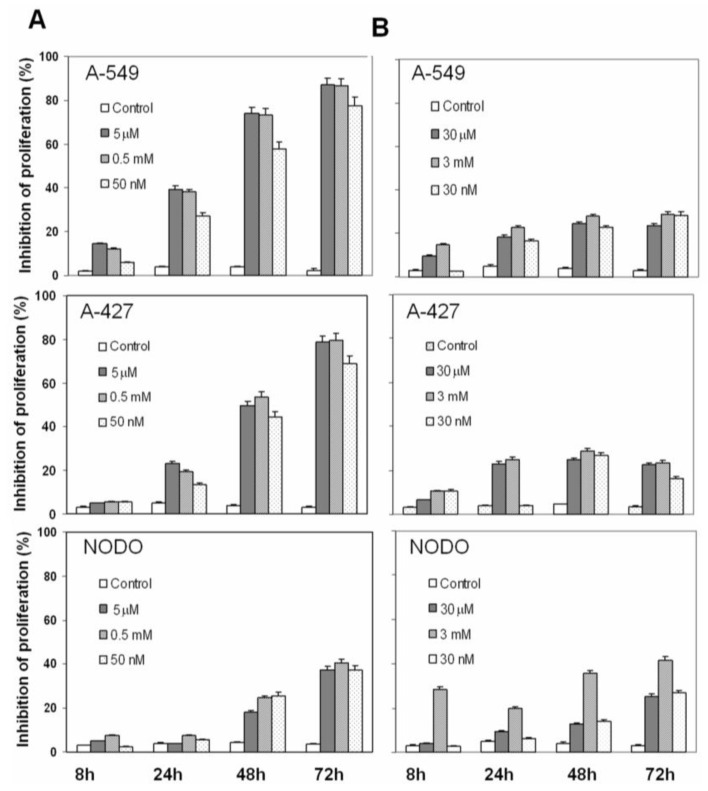
Effect of plasma concentrations of Pac (**A**) and Car (**B**) on NSCLC cell lines. Cell proliferation was estimated using the MTT assay, as described in “Methods”. Values are means ± SD of six measurements in four separate experiments.

Inhibition of proliferation by plasma concentrations of Car, Pac and both Car-Pac in NSCLC cells was investigated. In A-549 cells, Pac induced a time- and concentration-dependent inhibition of proliferation of 39.4%, 74.1% and 86.9% at 24 h, 48 h and 72 h, respectively. Low doses of Pac induced a minor rate of proliferation inhibition (27.4%, 58.1% and 77.7% at the same times). By contrast, high doses of Pac induced a similar rate of proliferation inhibition as the plasma concentrations (38.2%, 73.3% and 86.4%; see Figure 1A). Similar effects were observed in A-427 cells (Figure 1A). However, NODO cells showed a greater resistance to Pac activity. As shown in Figure 1A, Pac plasma concentrations induced only a 3.8%, 18.1% and 37.5% inhibition of proliferation at 24 h, 48 h and 72 h, respectively (Figure 1A).

Car plasma concentrations also induced a time- and concentration-dependent inhibition of proliferation, but this inhibition was much less than Pac. As shown in Figure 1B, A-549 and A-427 cells showed similar proliferation inhibition at 48 h (24.4% and 24.9%, respectively), while in NODO cells, this inhibition was 12.7%. At 72 h, all lung cancer cells showed similar proliferation inhibition (23.7% 22.5% and 25.4% in A-549, A-427 and NODO cells, respectively). Similar rates of proliferation inhibition were found with high and low doses of Car in A-549 and A-427 cells (Figure 1B). By contrast, high doses of Car at 72 h caused a high proliferation inhibition in NODO cells (41.7%; see Figure 1B).

**Figure 2:**
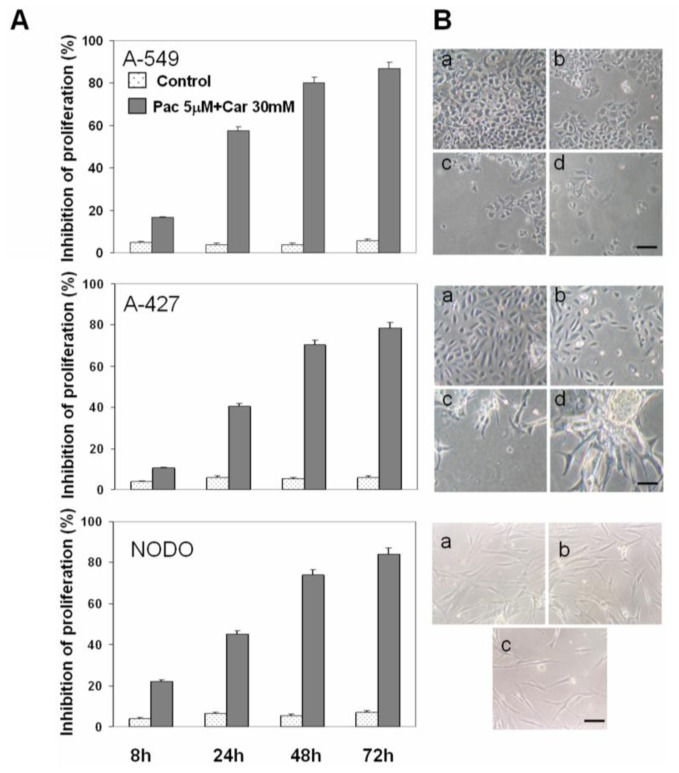
Effect of plasma concentrations of the Car-Pac combination on NSCLC cell lines. (**A**) Cell proliferation estimated by the MTT assay, as described in “Methods”. Values are means ± SD of six measurements in four separate experiments; (**B**) Representative phase-contrast micrographs showing morphological changes observed in NSCLC cell lines after drug exposure at 8 h (**a**), 24 h (**b**), 48 h (**c**) and 72 h (**d**). Scale bar: 100 μm.

The combination of plasma concentrations of Car and Pac had a cumulative effect at 24 h in A-549 and A-427 cells. The effect of these concentrations reached a plateau at 72 h by saturation of the mechanisms of cell death (Figure 2). By contrast, in NODO cells, the Car-Pac combination enhanced the proliferation inhibition (45.3%, 74% and 84.3% at 24 h, 48 h and 72 h, respectively) more than the sum of the effect of both drugs used independently (Figure 2).

### 2.2. Apoptosis Analysis

**Figure 3:**
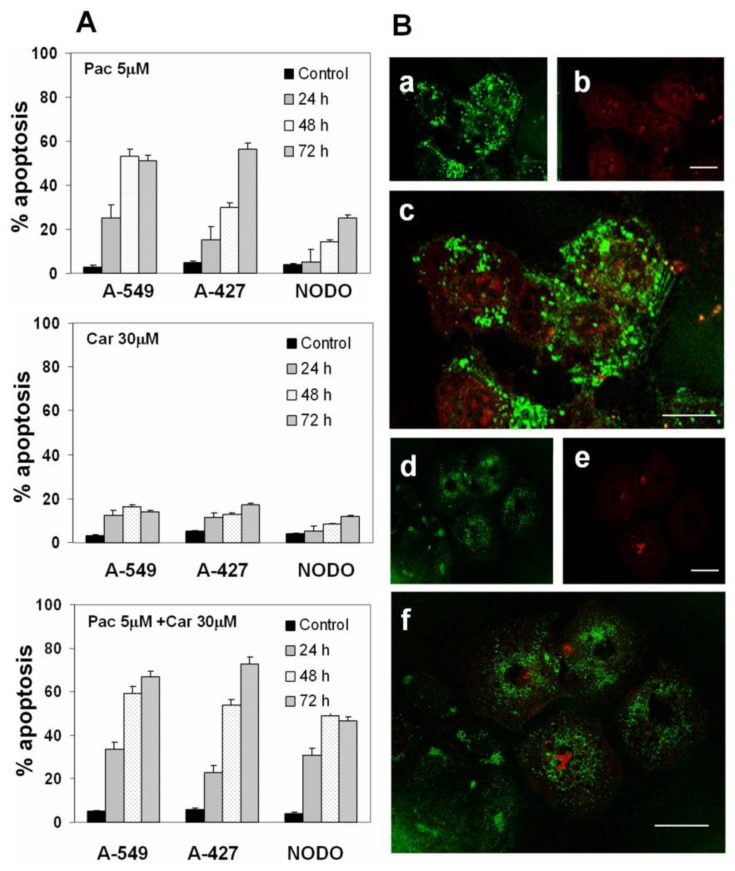
Apoptosis induced by plasma concentrations of drugs in NSCLC cell lines. (**A**) Percentage of apoptosis induced by Pac, Car and Pac-Car combination therapy. Values represent means ± SD of quadruplicate cultures; (**B**) To confirm apoptotic induction, cells were analyzed using annexin V-FITC staining and confocal microscopy. The figure shows representative images of stronger staining in A-549 cells (**a**: annexin V-FITC staining; **b**: cell nuclei counterstained with PI; **c**: overlay) and weak staining in NODO cells (**d**: annexin V-FITC staining; **e**: cell nuclei counterstained with PI; **f**: overlay). The study was carried out at 48 h. Scale bar: 25 μm.

Plasma concentrations of Pac induced apoptosis at rates of 25.3%, 53.3% and 51.3% in A-549 and at 15.4%, 30.2% and 56.6% in A-427 cells at the times analyzed (24 h, 48 h and 72 h). However, metastasis-derived NODO cells showed significantly lower rates of apoptosis (5.1%, 14.3% and 25.4% at the same times; see Figure 3A). Carboplatin at plasma concentrations induced a lower level of apoptosis than Pac in all cell lines, especially at the more prolonged exposure times (16.3% and 14.1% in A-549 cells and 12.8% and 17% in A-427, at 48 h and 72 h, respectively). The lower percentage of apoptosis was found in NODO cells. Car-Pac in combination produced a significant increase in percent apoptosis in all cell lines. Rates of apoptosis of 66.7%, 72.6% and 46.5% were reached in A-549, A-427 and NODO cells, respectively, at 72 h of drug exposure (Figure 3A). Apoptosis induction was confirmed using confocal laser-scanning microscopy (Figure 3B).

### 2.3. Modulation of *MDR1* and *MRP3* Gene Expression in NSCLC Cell Lines

**Figure 4:**
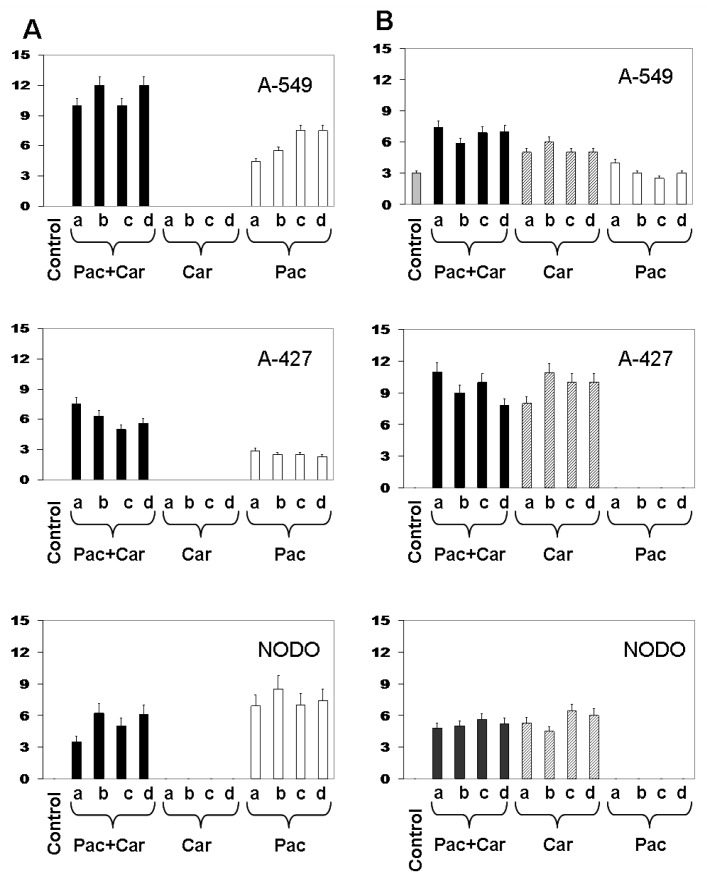
Expression of the MDR1 (**A**) and MRP3 (**B**) resistance gene in A-549, A-427 and NODO cell lines. Figures show quantification of gene expression by real time PCR. Cells were exposed to plasma concentrations of Pac, Car and Pac-Car at 8 h, 24 h, 48 h and 72 h (**a**, **b**, **c** and **d**, respectively). Parental cells without drug exposure were assessed as controls. Values are means ± SD of four separate experiments.

Expression of resistance genes was detected in NSCLC cell lines before and after drug treatment. A-549 cells showed MDR1 relative expression of 4.4 ± 0.3 after 8 h of Pac plasmatic concentration exposure, which increased to 7.5 ± 0.4 after 72 h after exposure (Figure 4A). NODO cells showed MDR1 relative expression of 6.9 ± 0.3 at 8 h and 7.4 ± 0.4 at 72 h. By contrast, A-427 cells showed relative expression of only 2.9 ± 0.1 at 8 h after exposure without significant changes at the different times analyzed. Carboplatin did not induce any modulation of MDR1 expression of the three lines (Figure 4A). Interestingly, combined Pac-Car treatment induced a greater MDR1 expression than the Pac exposure alone, which was particularly significant in A-549 (10 ± 0.2 at 8 h and 12 ± 0.8 at 72 h) and in A-427 cells (7.5 ± 0.3 at 8 h and 5.6 ± 0.3 at 72 h). NODO cells showed a low increase in the relative *MDR1* expression with 3.5 ± 0.3 at 8 h and 6.1 ± 0.5 at 72 h after exposure (Figure 4A). By contrast, Pac did not modulate *MRP3* expression, while Car induced a slight increase of MRP3 expression in A-549, A-427 and NODO cell lines. These modulations were maintained over time. Unlike the case of MDR1, the Pac-Car combination did not enhance MRP3 expression in NSCLC cell lines (Figure 4B).

## 3. Discussion

The association of Pac and Car, two powerful drugs used in the adjuvant therapy of NSCLC patients [15,16], showed a statistically significant improvement of overall and disease-free survival [17]. However, the number of patients who benefit from this adjuvant chemotherapy is low due to the development of MDR. A significant association between the expression of resistance genes, treatment failure and a decrease in overall survival has been clearly demonstrated in lung cancer patients, supporting the prognostic implication of these genes [13,18]. The cellular mechanism of this resistance may be multifactorial, including decreased drug accumulation in the cytoplasm, increased intracellular detoxification and increased DNA repair capacity. To date, the exact mechanism by which Pac and Car induce cell resistance in lung cancer cells is not fully understood.

Contradictory articles have been published for *MDR* gene expression. Previous studies showed that both vinca alkaloids and taxanes are good substrates of P-gp [19] and that cell lines exposed to drugs such as vincristine or Pac showed resistance associated with the expression of MDR1 [20]. By contrast, MRP is an efficient transporter of vinca alkaloids, but not taxanes [21]. Recently, other resistance mechanisms, such as ABCB5, an ATP-binding cassette (ABC) transporter protein, have been linked to Pac and Docetaxel resistance [22]. In human oesophageal squamous cancer cells, up-regulation of P-gp is involved in increased Pac resistance, but not in cisplatin resistance [23]. The connection between increased *MDR1* gene expression and Pac has also been described in resistant human colon cancer (DLD1) and glioblastoma (U87) cell lines, while *MRP1* expression decreased [24]. Array studies by [25] showed that the upregulation of the *MDR1* gene is the dominant mechanism of Pac and vincristine resistance in the breast cancer cell line MCF-7, suggesting that resistant cells exhibit different gene expression patterns depending on the drug treatment. *In vivo* studies using lung cancer xenograft models showed that drugs may induce resistance mediated by LRP, *MDR1* and *MRP* genes [26], the main candidates to explain treatment failure in NSCLC patients [27,28]. However, some authors conclude that the response to chemotherapy with Pac in NSCLC patients is related to MDR1 expression, but not to LRP expression [29]. By contrast, some authors found no relationship between expression of LRP, MDR1 or MRP1 and resistance in NSCLC [3,26]. In fact, Shimomura *et al.* showed similar ABCB1 mRNA levels in NSCL sensitive and resistant cell lines [30]. Our results clearly showed that the plasma concentration of Pac induced a 7.5-fold relative increase of MDR1 expression in A-549 and NODO cells. This increase was somewhat lower (three-fold) in A-427 cells. No modulation of MRP3 expression was found in any cell line. These results support a recent study by Maraz *et al.* who demonstrated that MDR1 expression is an efficient marker in lung cancer patients to determine their response to Pac [31]. This correlation has been confirmed in ovary cancer [32]. In addition, previous studies showed that cell lines resistant to Car and cisplatin showed increased efflux of the two dugs [33]. *In vitro* studies using lung cancer cells showed an induction of MDR1 and MRP expression by Car [34]. Our results clearly showed that Car increased levels of only MRP3 in the three lung cancer cell lines tested and not those of MDR1. The most important finding was that the Car-Pac combined treatment induced MDR1 and MRP3 overexpression with enhanced MDR1 expression, despite Car not having an effect on the MDR1 gene. These results suggest that adjuvant therapy in NSCLC patients using both drugs may not only induce, but also enhance, this resistance mechanism.

## 4. Experimental Section

### 4.1. Cell Culture

The lung carcinoma A-549 (CCL185) and A-427 (HTB-53) cell lines were obtained from ATCC and grown with Ham’s F12K and DEMEM medium, respectively (Sigma Chemical Co., St. Louis, MO, USA), supplemented with 10% heat-inactivated foetal bovine serum (FBS), 40 mg/L gentamycin and 500 mg/L ampicillin (Antibioticos S.A., Spain). Cells were maintained in a monolayer culture at 37 °C in an atmosphere containing 5% CO_2_. The NODO cell line (32050004) was developed from a lung cancer metastasis (Red de Bancos de Tumores, Granada, Spain) and was grown in an RPMI 1640 medium supplemented with 1% ITS (insulin-transferrin-selenium).

### 4.2. *In Vitro* Cell Proliferation Assay

Cells were seeded in 24-well plates (10^4^ cells per well). MTT [3-(4,5-dimethylthiazol-2-yl)-2, 5-diphenyltetrazolium bromide] solution (5 mg/mL) was added to each well (20 μL) and incubated for 4 h at 37 °C. After the medium was removed, 200 μL of dimethylsulfoxide (DMSO) was added to each well. Optical density was determined using a Titertek multiscan colorimeter (Flow, Irvine, CA, USA) at 570 nm and 690 nm. The proliferation effect of Car, Pac and the Car-Pac combination was determined for 72 h. Plasma concentrations of Pac (5 μM) and Car (30 μM) [35,36], and higher and lower plasma concentrations of the drugs, were used for the assays. Morphological changes were analyzed by optical microscopy.

### 4.3. Apoptosis Analysis

For analysis of the cell-cycle distribution, cells were harvested, washed twice with sample buffer (100 mg glucose; 100 mL PBS without Ca^2+^ or Mg^2+^), and fixed in 70% (*v*/*v*) cold ethanol for at least 1 h before staining. The cells were pelleted, washed once with sample buffer and resuspended in propidium iodide (PI) solution (50 μg/mL PI, 0.5 mg/mL RNase in sample buffer, pH 7.4) for 30 min in the dark. A fluorescence-activated cell sorter analysis was performed at 24 h, 48 h and 72 h after treatment. The data were collected and analyzed using the Cellfit program with a FACScan flow cytometer (Becton Dickinson, San Jose, CA, USA). To confirm apoptosis, cells were washed twice with PBS and incubated in binding buffer containing annexin V-FITC (25 μg/mL) and PI (25 μg/mL) in the dark for 15 min at room temperature (Annexin V-FITC Apoptosis Detection Kit I; BD Pharmingen, San Diego, CA, USA). Alternatively, the fluorescence was detected by confocal microscopy using a Leica DMI6000 microscope (Heidelberg, Germany).

### 4.4. RNA Extraction and Quantitative Real-Time PCR

Total RNA was extracted from cells using the Rneasy Mini kit (Qiagen). cDNA was generated using the Promega Reverse Transcription System (Promega, Madrid, Spain) using total cellular RNA (1 μg). Polymerase chain reaction (PCR) amplification of the resistance genes was performed using primers specific to MDR1 [37] and MRP3 [10]. RNA integrity was assessed by amplification of β-actin mRNA [37]. PCR products were analyzed by standard agarose gel electrophoresis (data not shown). In order to quantify resistance gene expression, TaqMan quantitative real-time PCR was performed using cDNA corresponding to 40 ng RNA per reaction. Primers specific for MDR1, MRP3 and β*-actin* and TaqMan Fast Mastermix (Applied Biosystems) were used and amplifications carried out on the Applied Biosystem 7500 (Applied Biosystems, CA, USA). Gene expression levels were calculated by the Δ*C*t method: Δ*C*t = mean value *C*t (mRNA reference) − mean value *C*t (mRNA of interest). Normalised Δ*C*T (delta cycle threshold) values were obtained by subtracting the β*-actin C*t from the gene of interest *C*t. The relative expression of mRNA of the gene of interest corresponded to the 2^Δ^*^C^*^t^ value.

### 4.5. Statistical Analysis

Statistical analyses were performed using the SPSS statistical software, version 16.0 (SPSS Inc., Chicago, IL, USA). The results were compared using the student’s *t*-test. All data are expressed as mean ± SD. Differences were considered statistically significant at a *p* value of less than 0.05.

## 5. Conclusions

In the present study, we used lung cancer culture cells to analyze the resistance mechanism induced by the use of the Car-Pac combination therapy. Our results clearly indicate that Pac induced resistance mediated by the *MDR1* gene and that Car induced the expression of the *MRP3* gene. However, the use of both drugs enhanced the resistance mediated by MDR1. Since Car-Pac is the main adjuvant therapy in NSCLC patients, these results may be clinically important to the treatment response. Future clinical validation will be necessary to confirm these findings.
